# Influence of assisted outpatient living in people with intellectual disabilities on individual quality of life and resilience – design of a doctoral thesis

**DOI:** 10.1192/j.eurpsy.2022.1560

**Published:** 2022-09-01

**Authors:** V. Raczkiewicz, A. Neumann, B. Buchberger

**Affiliations:** Universität Duisburg-Essen, Health Care Management And Research, Essen, Germany

**Keywords:** intellectual disability, assisted outpatient living, resilience, Quality of Life

## Abstract

**Introduction:**

The research field of disability and mental health is politically and socially relevant, because the psychological well-being of people with intellectual disabilities has often been trivialized in therapy and legislation. Since the pursuit of mental health is coming into focus due to removal of stigma and emerging awareness, people with disabilities must have equal opportunities to choose their place of residence and get suitable psychosocial support.

**Objectives:**

Our aim is to investigate the influence of assisted outpatient treatment (AOT) on the quality of life and resilience of people with intellectual disabilities who are living self-determined.

**Methods:**

A participatory mixed-methods design is chosen as it enables the greatest possible standardization and allows a high flexibility. The project will be divided into three parts: A systematic literature search to gain knowledge about the field and to estimate the sample size, a pre-post-comparison of the WHOQOL-BREF to evaluate AOT in terms of self-perceived quality of life and a focus-group of handicapped people to reflect the study results with attention on resilience.

**Results:**

To foster the doctoral thesis, several questions can be discussed: 1) How is the relation of disability and mental health to be described? 2) What might be the pros and cons of self-determined living? 3) Which steps need to be taken to implement AOT more often?

**Conclusions:**

The topic is relevant in the public health sector and the results could help to sensitize professionals and the general society regarding to participation in everyday life. The recommendations developed may serve to implement comparable forms of housing.

**Disclosure:**

No significant relationships.

